# SMTP-44D Inhibits Atherosclerotic Plaque Formation in Apolipoprotein-E Null Mice Partly by Suppressing the AGEs-RAGE Axis †

**DOI:** 10.3390/ijms24076505

**Published:** 2023-03-30

**Authors:** Michishige Terasaki, Keita Shibata, Yusaku Mori, Tomomi Saito, Takanori Matsui, Makoto Ohara, Tomoyasu Fukui, Keiji Hasumi, Yuichiro Higashimoto, Koji Nobe, Sho-ichi Yamagishi

**Affiliations:** 1 Department of Medicine, Division of Diabetes, Metabolism, and Endocrinology, Showa University School of Medicine, 1-5-8 Shinagawa, Tokyo 142-8666, Japan; 2 Division of Pharmacology, Department of Pharmacology, Toxicology and Therapeutics, School of Pharmacy, Showa University, 1-5-8 Hatanodai, Shinagawa-ku, Tokyo 142-8555, Japan; 3 Pharmacological Research Center, Showa University, 1-5-8 Hatanodai, Shinagawa-ku, Tokyo 142-8555, Japan; 4 Department of Medicine, Division of Diabetes, Metabolism, and Endocrinology, Anti-Glycation Research Section, Showa University School of Medicine, 1-5-8 Shinagawa, Tokyo 142-8666, Japan; 5 Department of Pathophysiology and Therapeutics of Diabetic Vascular Complications, Kurume University School of Medicine, Kurume 830-0011, Japan; 6 TMS Co., Ltd., KeioFuchu1chome Bldg. 11F, 1-9 Fuchucho, Fuchu-shi, Tokyo 183-0055, Japan; 7 Department of Applied Biological Science, Tokyo University of Agriculture and Technology, 3-5-8 Fuchu-shi, Tokyo 183-8509, Japan; 8 Department of Chemistry, Kurume University School of Medicine, Kurume 830-0011, Japan

**Keywords:** SMTP-44D, atherosclerosis, macrophages, AGEs, RAGE, Cdk5, CD36

## Abstract

SMTP-44D has been reported to have anti-oxidative and anti-inflammatory reactions, including reduced expression of receptor for advanced glycation end products (RAGE) in experimental diabetic neuropathy. Although activation of RAGE with its ligands, and advanced glycation end products (AGEs), play a crucial role in atherosclerotic cardiovascular disease, a leading cause of death in diabetic patients, it remains unclear whether SMTP-44D could inhibit experimental atherosclerosis by suppressing the AGEs–RAGE axis. In this study, we investigated the effects of SMTP-44D on atherosclerotic plaque formation and expression of AGEs in apolipoprotein-E null (*Apoe^−/−^*) mice. We further studied here whether and how SMTP-44D inhibited foam cell formation of macrophages isolated from *Apoe^−/−^* mice ex vivo. Although administration of SMTP-44D to *Apoe^−/−^* mice did not affect clinical or biochemical parameters, it significantly decreased the surface area of atherosclerotic lesions and reduced the atheromatous plaque size, macrophage infiltration, and AGEs accumulation in the aortic roots. SMTP-44D bound to immobilized RAGE and subsequently attenuated the interaction of AGEs with RAGE in vitro. Furthermore, foam cell formation evaluated by Dil-oxidized low-density lipoprotein (ox-LDL) uptake, and gene expression of *RAGE, cyclin-dependent kinase 5* (*Cdk5*) and *CD36* in macrophages isolated from SMTP-44D-treated *Apoe^−/−^* mice were significantly decreased compared with those from saline-treated mice. Gene expression levels of *RAGE* and *Cdk5* were highly correlated with each other, the latter of which was also positively associated with that of *CD36*. The present study suggests that SMTP-44D may inhibit atherosclerotic plaque formation in *Apoe^−/−^* mice partly by blocking the AGEs-RAGE-induced ox-LDL uptake into macrophages via the suppression of Cdk5-CD36 pathway.

## 1. Introduction

Advanced glycation end products (AGEs), whose formation and accumulation are increased under hyperglycemic and/or oxidative stress conditions [1,2,3], have been considered to play a crucial role in the pathogenesis of development and progression of atherosclerotic cardiovascular disease, one of the leading causes of death in developed countries [4,5,6]. AGEs not only alter structural and functional properties of extracellular matrix proteins, such as collagen and laminin, but also evoke oxidative stress and inflammatory and thrombotic reactions in various cell types, including macrophages through the interaction with their cell surface receptor for AGEs (RAGE) [1,7,8,9,10,11]. Indeed, AGEs are localized in macrophage-derived foam cells within the atherosclerotic plaques and promote the foam cell formation of macrophages, thereby being involved in atherosclerotic plaque instability in high-risk patients [12,13,14,15,16,17]. Circulating and tissue-accumulated levels of AGEs are associated with the increased risk of cardiovascular disease and death in both diabetic and non-diabetic subjects [18,19,20,21,22]. Further, inhibition of AGEs-RAGE interaction by administration of a recombinant soluble form of RAGE or knockout of *RAGE* gene has been reported to inhibit the development and progression of atherosclerosis in apolipoprotein-E null (*Apoe^−/−^*) mice [23,24,25]. These observations suggest that blockade of the AGEs–RAGE axis in macrophages may be a novel therapeutic target for atherosclerotic cardiovascular disease.

SMTP is a family of small-molecule triprenyl phenol metabolites derived from the fungus *Stacybotrys microspora* [26,27]. Among them, SMTP-7, which exhibits pro-thrombolytic, anti-inflammatory, and anti-oxidative activities, has been shown to be effective in treating several animal models of cerebral infarction in rodents and monkeys [28,29,30,31,32,33,34,35] and two types of acute kidney injury models in mice [36,37]. Furthermore, SMTP-44D, an analog of SMTP-7, has also been reported to exert neuroprotective effects in cell culture and animal models through anti-inflammatory and anti-oxidative properties [38,39,40]. Indeed, SMTP-44D inhibits inflammatory reactions, including *RAGE* expression in, and apoptotic cell death of, high-glucose-exposed Schwann cells via its anti-oxidative property [40]. Moreover, SMTP-44D has improved allodynia and restored the decreased blood flow and conduction velocity of sciatic nerve in a type 1 diabetic animal model [41]. However, the effects of SMTP-44D on the progression of atherosclerosis in *Apoe^−/−^* mice remain to be elucidated. 

Esterification of free cholesterol in macrophages and subsequent foam cell formation in the subendothelial space is one of the initial characteristic features of atherosclerosis [42,43]. The macrophage scavenger receptor, CD36, has been shown to accelerate the uptake of oxidized low-density lipoprotein (ox-LDL), thereby promoting foam cell formation within the atherosclerosis [44]. We have previously found that cyclin-dependent kinase 5 (Cdk5) is involved in CD36-mediated foam cell formation of macrophages in vitro, which process is stimulated by AGEs through the interaction with RAGE [45,46]. Therefore, in this study, we investigated the effects of SMTP-44D on atherosclerotic plaque formation and expression of AGEs in *Apoe^−/−^* mice. We further studied here whether SMTP-44D inhibited foam cell formation of macrophages isolated from *Apoe^−/−^* mice by inhibiting the Cdk5-CD36 pathway though suppression of the AGEs–RAGE axis. 

## 2. Results

### 2.1. Clinical Characteristics of Apoe^−/−^ Mice

Laboratory data of *Apoe^−/−^* mice injected with SMTP-44D at 30 mg/kg/day or saline every other day intraperitoneally are presented in Table 1. There were no significant differences in food intake, body weight, systolic blood pressure (SBP), diastolic blood pressure (DBP), heart rate, total-cholesterol (Total-C), high-density lipoprotein cholesterol (HDL-C), triglycerides, insulin, fasting blood glucose (FBG), or circulating AGEs levels between two groups.

### 2.2. Effects of SMTP-44D on Atherosclerotic Lesions, Macrophage Infiltration, and AGEs Accumulation in the Aortic Roots in Apoe^−/−^ Mice

**Figure 1:**
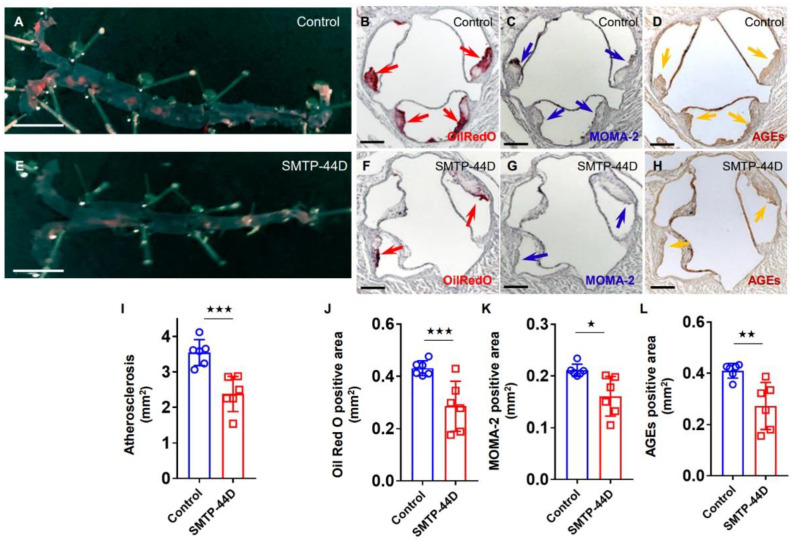
Effects of SMTP-44D on atherosclerotic lesions, macrophage infiltration, and accumulation of AGEs in the entire aorta and aortic roots in *Apoe^−/−^* mice administered with SMTP-44D or saline. (**A**–**H**) Representative images are shown. The aortic surface was stained with Oil red O. White scale bars correspond to a length of 5 mm. (**A**,**E**), while the aortic roots were stained with Oil red O (**B**,**F**), MOMA-2 (**C**,**G**), and AGEs (**D**,**H**). Magnification x40. Black scale bars correspond to a length of 200 μm. Surface area of the atherosclerotic lesions (**I**), and the cross-sectional area of atheromatous plaque size (**J**), macrophage infiltration (**K**), and AGEs accumulation (**L**) in the aortic roots were quantified and shown as mean ± standard deviation. Number = 6 for each group. ^★★★^ *p* < 0.005, ^★★^ *p* < 0.01, and ^★^ *p* < 0.05 vs. saline-treated mice.

We examined the effects of intraperitoneal injection of SMTP-44D for 4 weeks on atherosclerotic plaque formation in both the entire aorta and the cross section of the aortic roots derived from *Apoe^−/−^* mice. As shown in Figure 1, administration of SMTP-44D to the mice for 4 weeks significantly decreased the surface area of atherosclerotic lesions (Figure 1A,E,I) and reduced the atheromatous plaque size (Figure 1B,F,J), macrophage infiltration evaluated by MOMA-2 staining (Figure 1C,G,K), and AGEs accumulation (Figure 1D,H,L) in the aortic roots, compared with those of saline-treated mice.

### 2.3. Binding of SMTP-44D to Immobilized RAGE and Its Effect on AGEs–RAGE Interaction

**Figure 2:**
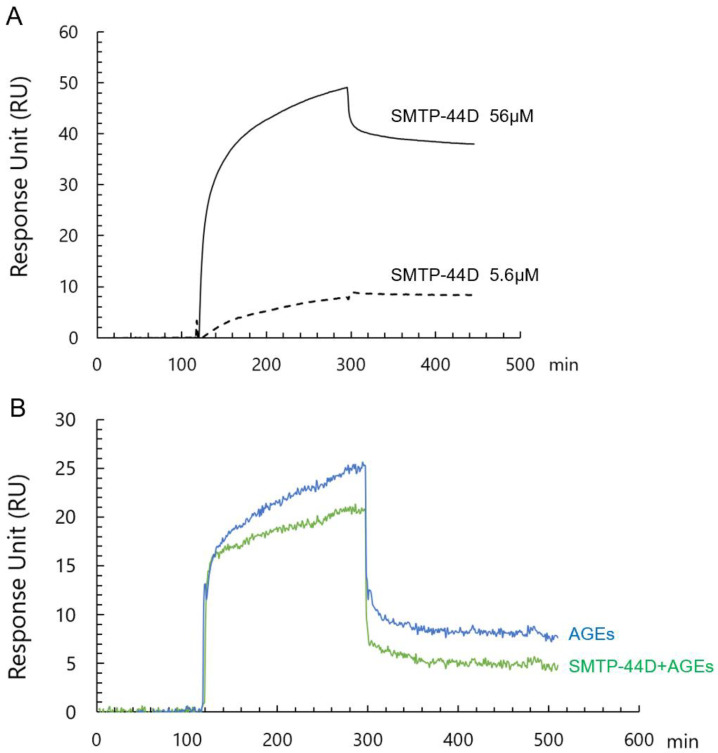
Interaction of SMTP-44D at 56 μmol/L and 5.6 μmol/L to immobilized RAGE was analyzed by bio-layer interferometry using BIAcore system (**A**). Effects of 56 μmol/L SMTP-44D on the interaction of 250 μg/mL AGEs with immobilized RAGE were evaluated (**B**). The binding protein is expressed as RU.

We then examined whether SMTP-44D bound to immobilized RAGE by surface plasmon resonance. Bio-layer interferometry analysis using the BIAcore system revealed that SMTP-44D bound to RAGE with a dissociation constant (*K*_d_) of 25 μmol/L (Figure 2A). When 56 μmol/L SMTP-44D was added into the wells coated with immobilized RAGE in the presence of 250 μg/mL AGEs, it inhibited the binding of AGEs to RAGE by about 20% (Figure 2B).

### 2.4. Effects of SMTP-44D on ox-LDL Uptake into, and RAGE, CD36, and Cdk5 Gene Expression in, Peritoneal Macrophages Isolated from Mice

**Figure 3:**
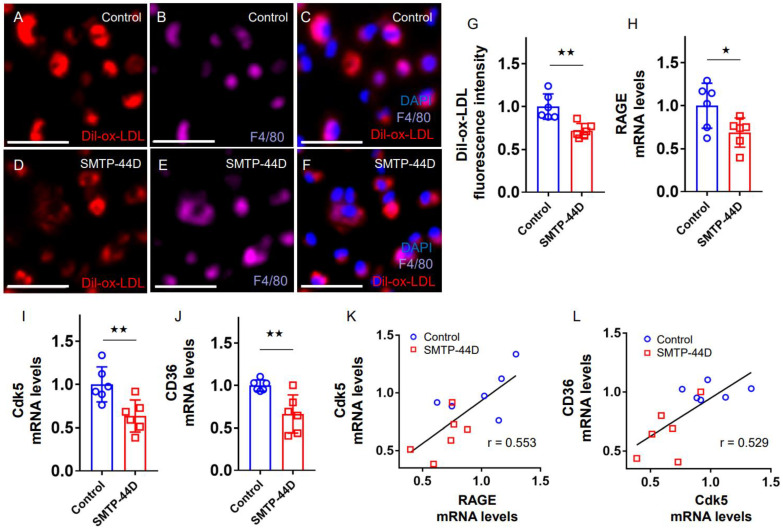
Effects of SMTP-44D on Dil-ox-LDL uptake into, and *RAGE*, *Cdk5*, and *CD36* gene expression in, *Apoe^−/−^* mice. Peritoneal macrophages were extracted from *Apoe^−/−^* mice injected with SMTP-44D at 30 mg/kg/day or saline every other day for 4 weeks. (**A**–**F**) Representative immunofluorescent staining images in peritoneal macrophages. Dil-ox-LDL positive cells were stained in red (**A**,**D**), while F4/80 were in purple (**B**,**E**). (**C**,**F**) Merge images. Scale bars, 50 μm. Quantification of fluorescence intensity in red. Dil-ox-LDL uptake was shown as a relative value compared to control mice (**G**). Gene expression levels of *RAGE* (**H**), *Cdk5* (**I**), and *CD36* (**J**) derived from *Apoe^−/−^* mice, and their correlation (**K**,**L**). Total RNAs were reverse-transcribed, and the resulting cDNAs were amplified by real-time PCR. Data were normalized by the intensity of *GAPDH* mRNA-derived signals and expressed as a relative to the control values. Number = 6 for each group. Error bars are standard deviation. ^★^ *p* < 0.05 and ^★★^ *p* < 0.01 vs. control.

We next investigated the effects of SMTP-44D on foam cell formation of macrophages extracted from *Apoe^−/−^* mice. Immunofluorescent staining revealed that 1,1’-dioctadecyl-3,3,3’,3’-tetramethylindocarbocyanine perchlorate-oxidized-low-density (Dil-ox-LDL)-positive cells were co-stained with F4/80 (Figure 3A–F), reflecting the uptake of ox-LDL into mouse peritoneal macrophages. Macrophage foam cell formation evaluated by Dil-ox-LDL uptake was significantly suppressed in SMTP-44D-treated mice compared with saline-treated mice (Figure 3G). Further, levels of *RAGE, Cdk5,* and *CD36* gene expression in peritoneal macrophages derived from SMTP-44D-treated *Apoe^−/−^* mice were significantly inhibited compared with those from saline-treated mice (Figure 3H–J). In addition, gene expression levels of *RAGE* and *Cdk5* were highly correlated with each other, the latter of which was also positively associated with that of *CD36* (Figure 3K,L).

## 3. Discussion

We have previously found that SMTP-44D has anti-oxidative, anti-inflammatory, and neuroprotective properties in experimental diabetic neuropathy and suppresses *RAGE* expression in immortalized mouse Schwann cells [38,39,40,41]. Although activation of RAGE with its ligands, AGEs has been known to play a crucial role in the development and progression of atherosclerotic cardiovascular disease, it remains unclear whether SMTP-44D could inhibit experimental atherosclerosis by suppressing the AGEs–RAGE axis. To address the issue, we first examined the effects of SMTP-44D on atherosclerotic plaque formation and AGEs expression in *Apoe^−/−^* mice. In this study, administration of SMTP-44D to *Apoe^−/−^* mice did not affect clinical or biochemical parameters; however, we found that SMTP-44D significantly decreased the surface area of atherosclerotic lesions and reduced atheromatous plaque size, macrophage infiltration, and AGEs expression in the aortic roots. Furthermore, we found for the first time that SMTP-44D bound to immobilized RAGE and subsequently attenuated the interaction of AGEs with RAGE. We have previously shown that AGEs–RAGE interaction evokes oxidative stress generation in various kinds of cells and tissues, which may further promote the formation and accumulation of AGEs [8,9,10,11,47,48,49,50]. Indeed, AGEs accumulation in the kidneys was reported to be suppressed in *RAGE*-deficient diabetic rats [51]. DNA aptamer raised against AGEs inhibited the binding of AGEs to RAGE in vitro and reduced the levels of AGEs in the kidneys of diabetic mice [52]. These observations suggest that there is a positive feedback loop between activation of RAGE downstream pathway and AGEs formation, which could partly explain why, although SMTP-44D administration did not affect circulating AGEs levels, it significantly reduced AGEs accumulation in the aortic roots in *Apoe^−/−^* mice. In other words, attenuation of the AGEs–RAGE interaction by SMTP-44D within the atherosclerotic plaques might contribute to the decreased levels of AGEs in the aortic roots in *Apoe^−/−^* mice. 

In the present study, Dil-ox-LDL uptake into, and *RAGE* gene expression of, macrophages isolated from SMTP-44D-treated *Apoe^−/−^* mice were significantly decreased compared with those from saline-treated mice. Recently, we have shown that DNA-aptamer raised against RAGE attenuated the interaction of AGEs to RAGE and subsequently inhibited the AGEs-induced ox-LDL uptake into macrophages [45]. Moreover, neutralizing an antibody or DNA aptamer raised against RAGE or an antioxidant prevents the AGEs-induced *RAGE* gene expression in a variety of cells [9,47,48,53], thus suggesting that AGEs–RAGE–induced oxidative stress generation might increase *RAGE* expression. Given that macrophage foam cell formation is one of the crucial steps of atherosclerosis [44], blockade of the interaction of RAGE with AGEs by SMTP-44D could cause the reduced *RAGE* gene expression in macrophages, which may lead to the decrease of atheromatous plaque size and macrophage infiltration in *Apoe^−/−^* mice.

**Figure 4:**
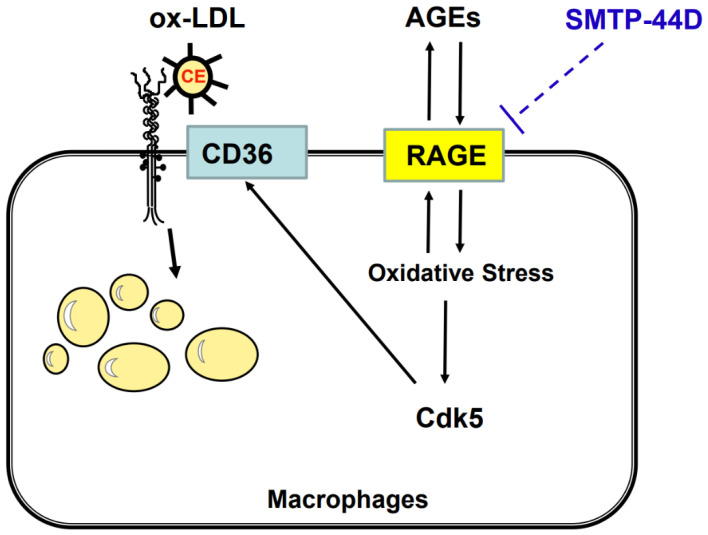
Possible anti-atherosclerotic actions of SMTP-44D. SMTP-44D could inhibit the AGEs-RAGE-induced macrophage foam cell formation through the suppression of the CD36-Cdk5 pathway. AGEs, advanced glycation end products; RAGE, receptor for AGEs; Cdk5, cyclin-dependent kinase 5; ox-LDL, oxidized low-density lipoprotein.

CD36 is one of the main scavenger receptors, which could promote macrophage foam cell formation in atherosclerosis [42,44]. On the other hand, Cdk5 is known to be a unique molecule because in contrast to other Cdk family members, it is not a modulator of cell cycle progression [54,55,56], but a regulator of gene regulation and cell survival [57]. Cdk5 is constitutively expressed in macrophages, which may contribute to enhancement of inflammatory reactions [55]. We have previously found that Cdk5 is involved in CD36-mediated foam cell formation of macrophages in vitro, which process is stimulated by AGEs through the interaction with RAGE [45,46]. In the present study, gene expression of *Cdk5* and *CD36* in macrophages isolated from SPTP-44D-treated *Apoe^−/−^* mice was significantly decreased compared with those from saline-treated mice. Further, gene expression levels of *RAGE* and *Cdk5* were highly correlated with each other, the latter of which was also positively associated with that of *CD36*. These findings suggest that SMTP-44D could inhibit the AGEs–RAGE-induced ox-LDL uptake into macrophages via the suppression of the Cdk5-CD36 pathway (Figure 4).

Our study has some potential limitations. First, providing the mRNA expression data of *RAGE*, *Cdk5*, and *CD36* may not be sufficient to definitely claim the involvement of the Cdk5-CD36 pathway in AGEs–RAGE-induced ox-LDL uptake by macrophages. However, we have already shown that (1) blockade of the interaction of AGEs with RAGE inhibits the ox-LDL uptake in AGEs-exposed macrophages by suppressing gene expression of *Cdk5* and *CD36*, (2) an inhibitor of Cdk5 reduces the AGEs-induced up-regulation of *CD36* mRNA levels in macrophages, (3) *CD36* gene expression and ox-LDL uptake are correlated with each other, and (4) anti-CD36 antibody attenuates the AGEs-induced ox-LDL uptake by macrophages [45,58]. Therefore, although we did not evaluate RAGE, Cdk5, and CD36 protein levels in the present study, these observations suggest that mRNA levels of *RAGE*, *Cdk5*, and *CD36* could reflect their protein expression levels and functionally associated with ox-LDL uptake into macrophages. Lacking samples, we could not evaluate these protein expression levels, which will make the mechanism concrete. Second, we did not evaluate Cdk5 activity levels. However, there are some papers to show that Cdk5 activity is highly associated with *Cdk5* gene expression levels [59,60]. Third, although we did not measure oxidative stress in macrophages in this study, we have already reported that an antioxidant mimicked the effects of RAGE aptamer in macrophages; it significantly decreased the AGEs-induced macrophage foam cell formation via inhibition of the Cdk5-CD36 pathway [45]. Fourth, the data presented here in most of the experiments were statistically significant, but their effects may be modest. However, we showed here that SMTP-44D significantly decreased the surface area of atherosclerotic lesions and reduced atheromatous plaque size, macrophage infiltration, and AGEs expression in the aortic roots of atherosclerotic animal model by attenuating the interaction of AGEs with RAGE. Moreover, the mechanistic study suggests that Cdk5-CD36 pathway could be a molecular target of SMTP-44D in AGEs–RAGE-induced ox-LDL uptake by macrophages. Therefore, our present study has a potential therapeutic advancement to treat atherosclerosis and may also provide a novel strategy for preventing various types of AGEs-related aging disorders.

## 4. Materials and Methods

### 4.1. Materials and Reagents

SMTP-44D [40,41] was produced by TMS Co., Ltd. (Tokyo, Japan). Dil-ox-LDL was purchased from Highland Technology Center (Frederick, MD, USA). Anti-F4/80 antibody (Alexa Fluor^®^ 647, Ab204467) was from Abcam (Cambridge, UK). Roswell Park Memorial Institute (RPMI) 1640 medium from Sigma Aldrich (St. Louis, MO, USA), and vectashield mounting medium (H-1500) was purchased from Vector Laboratories (Burlingame, CA, USA).

### 4.2. Animal Experiments

The protocol and design of this experiments were permitted by the Animal Care Committee of Showa University (approval number: 04017). All sacrifices or surgeries were performed by general anesthesia using isoflurane and with efforts to minimize the suffering.

A total of 12 male *Apoe^−/−^* mice at 8 weeks old were purchased from Sankyo Labo Service (Tokyo, Japan). The mice were kept on a standard food with free access to water in the Division of Animal Experimentation of Showa University School of Medicine. The rooms were controlled at 21 °C temperature, under a 12 h dark and light cycle and 40–60% humidity. At 17 weeks old, the mice were fed an atherogenic diet containing 30% fat, 20% sucrose, 8% NaCl, and 0.15% cholesterol (Oriental Yeast, Tokyo, Japan) [61,62,63,64,65], and were randomly assigned to intraperitoneal injection with SMTP-44D at 30 mg/kg/day or saline every other day. At 21 weeks old, peritoneal macrophages were obtained from mice after intraperitoneal injection of thioglycolate broth for measuring Dil-ox-LDL uptake and gene expression. Then, blood samples were collected from the descending vena cava. After being perfused with PBS and then with 4% paraformaldehyde, the aorta was carefully excised from the root to abdominal area, and the entire aorta and cross sections of the aortic root were stained with Oil red O for evaluation of atherosclerotic lesions, as previously described [61,62,63,64,65]. To investigate macrophage infiltration or AGEs accumulation in aortic roots, cross sections were stained with the anti-mouse MOMA-2 antibody (Chemicon International Inc., Temecula, CA, USA) [61,62,63,64,65] or AGEs [66]. The degree of atherosclerotic lesions, macrophage infiltration, and AGEs accumulation were measured by image analyzer as previously described (NIH Scion Image, Frederick, MD, USA) [61,62,63,64,65].

### 4.3. Characteristics and Biochemical Parameters in Mice 

After 12-h fasting, blood samples were collected and then used for the measurement of biochemical parameters. Body weight, food intake, systolic and diastolic blood pressure (SBP and DBP), and heart rate were calculated, and triglycerides, total-cholesterol (Total-C), high-density lipoprotein cholesterol (HDL-C), fasting blood glucose (FBG) and insulin were measured as described previously [46,58,61,62,63,64,65,67]. Serum levels of AGEs were measured by a competitive ELISA using polyclonal antibodies raised against AGEs [68].

### 4.4. Biophysical Interaction Analysis

The affinity of SMTP-44D/AGEs to RAGE was assessed by association and dissociation phases using a BIAcore 1000 (GE Healthcare, Buckinghamshire, UK). SMTP-44D was injected to the flow cell at concentration of 56 μmol/L or 5.6 μmol/L at flow rate of 10 μL/min at 25 °C. The sensor chip was regenerated, then the control signals reflecting the bulk effect of buffer were subtracted from the assay curve, as previously described [69]. Equilibrium dissociation constant (*K*_d_) was detected by the equation for 1:1 Langmuir binding, and mass change on the sensor tip evoked by binding between molecules was detected as a response unit (RU) [69].

### 4.5. Dil-ox-LDL Uptake into Mouse Macrophages 

Peritoneal macrophages were isolated from *Apoe^−/−^* mice using a peritoneal lavage with 8 mL of ice-cold PBS. They were suspended in a culture medium and seeded onto 3.5 cm dishes. After 1 h of incubation, the adherent macrophages were treated with 10 µg/mL Dil-ox-LDL in RPMI 1640 medium containing anti-F4/80 antibody and 10% FCS in 5% CO_2_ at 37 °C for 18 h as described previously [46,58,67]. After washing with PBS, they were mounted in a Vectashield mounting medium (H-1500), and immunofluorescence was measured by using Keyence BZ-X710 microscope and BZ-X800 software (Osaka, Japan). Then, the fluorescent intensity of red color per cells was quantified [45,46,58,67]. 

### 4.6. Levels of Gene Expression 

Adherent macrophages were used for evaluating gene expression. Gene expression levels were analyzed by quantitative real-time RT-PCR using the gene expression assay of SYBR Green or TaqMan based gene expression assay as described previously [46,58,61,62,63,64,65,67]. In brief, total RNA was normalized from the *Apoe^−/−^* mice to synthesize cDNA. Values of gene expression were normalized by the intensity of glyceraldehyde 3-phosphate dehydrogenase (GAPDH) mRNA-derived signals, then the data were expressed as a relative to the controls. Probes and primers were as follows: mouse; *RAGE*, Mm00545815_ml; *Cdk5*, NM_007688.4; *CD36*, Mm01135198_ml; *Gapdh,* Mm03302249_g1. 

### 4.7. Statistical Analysis

All data were expressed as mean ± standard deviation. Data were compared between two groups by unpaired t-test. The correlation between two groups was analyzed by Peason’s correlation test. All analyses were performed using PRISM software (version 7.05, GraphPad Inc., San Diego, CA, USA). A value of *p* < 0.05 was defined statistically significant.

## 5. Conclusions

The present study showed that SMTP-44D could inhibit atherosclerotic plaque formation in *Apoe^−/−^* mice partly by blocking the AGEs–RAGE-induced ox-LDL uptake into macrophages via the suppression of the Cdk5-CD36 pathway. Inhibition of the AGEs–RAGE axis by SMTP-44D in macrophages may be a novel therapeutic target for atherosclerotic disease.

## Figures and Tables

**Table 1:** Clinical characteristics of 21-week-old *Apoe^−/−^* mice injected with SMTP-44D or saline for 4 weeks.

	Saline	SMTP-44D
Number	6	6
Final body weight (g)	28.4 ± 2.2	29.4 ± 2.2
Food Intake (g/day)	4.0 ± 0.8	4.2 ± 0.7
SBP (mmHg)	103 ± 9	101 ± 10
DBP (mmHg)	60 ± 6	62 ± 7
Heart rate (bpm)	634 ± 58	650 ± 64
Total-C (mg/dL)	333 ± 100	383 ± 186
HDL-C (mg/dL)	23 ± 9	27 ± 8
Triglycerides (mg/dL)	53 ± 16	57 ± 8
Insulin (ng/mL)	0.39 ± 0.08	0.46 ± 0.14
FBG (mg/dL)	123 ± 14	121 ± 16
AGEs (μg/mL)	5.25 ± 1.32	5.38 ± 2.23

SBP, systolic blood pressure; DBP, diastolic blood pressure; Total-C, total-cholesterol; HDL-C, high-density lipoprotein cholesterol; FBG, fasting blood glucose; AGEs, advanced glycation end products; Results are presented as mean values ± standard deviation.

## Data Availability

The data in this study are available from the corresponding author upon reasonable request.
